# A Gene Responsible for Hybrid Incompatibility in Drosophila

**DOI:** 10.1371/journal.pbio.0020165

**Published:** 2004-06-15

Nearly 150 years after Darwin published *On the Origin of Species*, biologists are still debating how new species emerge from old—and even the definition of species itself. Darwin demurred from offering a hard and fast definition, suggesting that such a thing was “undiscoverable.” One of the more enduring definitions characterizes organisms as distinct reproductive units and species as groups of individuals that can interbreed and produce viable, fertile offspring. The lack of genetic exchange between species, called reproductive isolation, lies at the heart of this definition. Environmental changes can create physical barriers between populations that preclude mating between the populations. Reproductive isolation can also involve changes at the genetic level, when molecular barriers prevent two recently diverged populations from producing viable or fertile offspring. Such factors limit gene flow between diverging species and allow the emergence of genetically novel yet sound populations—that is, new species.[Fig pbio-0020165-g001]

**Figure pbio-0020165-g001:**
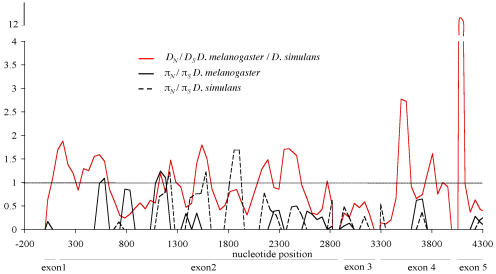
Multiple regions of *Hmr* show evidence for divergence driven by positive selection

At the heart of reproductive isolation is a phenomenon called hybrid incompatibility, in which closely related species are capable of mating but produce inviable or sterile offspring. The classic example of hybrid incompatibility is the male donkey–female horse cross, which yields a sterile mule, but many other cases have been documented among mammals, and thousands of plant crosses produce infertile offspring. Much has been learned about the genetic architecture of hybrid incompatibility by studying the offspring of closely related, or “sibling,” fruitfly species in the lab. Sibling species are morphologically very similar, or even indistinguishable, but typically do not interbreed in nature. In the lab, their offspring are either sterile or inviable, a fate that varies depending on the gender of the offspring and species of the parents. To elucidate the molecular mechanisms of reproductive isolation, biologists must first identify candidate hybrid incompatibility genes. Species- or lineage-specific functional divergence is an essential trait of these genes. (That is, the genes evolve different functions after the species diverge from their common ancestor.) While several such candidate genes have been identified in the fruitfly Drosophila melanogaster, none has been shown to display this functional divergence. Now, working with D. melanogaster and its sibling species D. simulans and D. mauritiana, Daniel Barbash, Philip Awadalla, and Aaron Tarone establish the functional divergence of a candidate hybrid compatibility gene and confirm its status as a true speciation gene.

Since the 1930s, investigations of reproductive isolation have been guided by the Dobzhansky-Muller model, which attributes hybrid incompatibility to the interactions between two or more genes that have evolved independently in two isolated populations. These independently evolving genes diverge functionally, and the interactions of these functionally divergent genes in a hybrid individual are responsible for the defective phenotypes observed (either inviability or sterility). If this is the case, the alleles, or versions, of the gene causing hybrid incompatibility should have distinct phenotypes in the two species. A corollary of the model says that the diverged allele *(A)* and not the ancestral allele *(a)* causes the incompatibility phenotype, which means that experimental manipulations of *A* but not *a* should affect the hybrid incompatibility phenotype.

Barbash et al. tested the model's predictions by genetically manipulating the alleles of the *Hybrid male rescue (Hmr)* gene from each sibling species and observing the mutations' effects on the flies' hybrid offspring. In previous experiments the researchers had shown that loss-of-function mutations in the D. melanogaster *Hmr* gene “rescue” hybrid individuals from the hybrid incompatibility phenotype (male inviability) typically observed in the offspring of crosses between D. melanogaster and its sibling species, and that increased *Hmr* activity suppresses rescue and kills hybrids. If D. melanogaster *Hmr* has functionally diverged between the species, then transgenes containing *Hmr* from sibling species should not cause the hybrid incompatibility phenotype caused by the D. melanogaster *Hmr*. The researchers tested this hypothesis by introducing transgenic *Hmr* genes from sibling species into D. melanogaster. In all cases, the hybrid male offspring of D. melanogaster/D. mauritiana and D. melanogaster/D. simulans crosses “were at least as viable as their brothers without the transgene.”

To examine this divergence at the genomic level, Barbash et al. compared the divergence of 250 genes in D. melanogaster and D. simulans and found that the *Hmr* gene was among the most rapidly evolving genes. By examining the frequency of mutations that have accumulated between D. melanogaster and sibling species relative to the number of mutations accumulated within species, the authors show that the mutations between species were by and large not neutral and that they occurred after D. melanogaster diverged from its sibling species, indicating that the gene has been under positive natural selection. Barbash et al. have not only identified a bona fide speciation gene by demonstrating its functional divergence, they've also created a platform for investigating the mechanisms through which such genes cause hybrid incompatibility and lay the groundwork for speciation.

